# Characterizing the supraspinal sensorimotor control of walking using MRI-compatible system: a systematic review

**DOI:** 10.1186/s12984-024-01323-y

**Published:** 2024-03-05

**Authors:** Yinglu Hong, Dapeng Bao, Brad Manor, Junhong Zhou

**Affiliations:** 1 School of Sport Medicine and Physical Therapy, Beijing Sport University, Beijing, China; 2 China Institute of Sport and Health Science, Beijing Sport University, Beijing, China; 3 Hebrew SeniorLife Hinda and Arthur Marcus Institute for Aging Research, Harvard Medical School, Boston, MA USA

**Keywords:** Gait regulation, MRI-compatible device, Supraspinal sensorimotor control

## Abstract

**Background:**

The regulation of gait is critical to many activities of everyday life. When walking, somatosensory information obtained from mechanoreceptors throughout body is delivered to numerous supraspinal networks and used to execute the appropriate motion to meet ever-changing environmental and task demands. Aging and age-related conditions oftentimes alter the supraspinal sensorimotor control of walking, including the responsiveness of the cortical brain regions to the sensorimotor inputs obtained from the peripheral nervous system, resulting in diminished mobility in the older adult population. It is thus important to explicitly characterize such supraspinal sensorimotor elements of walking, providing knowledge informing novel rehabilitative targets. The past efforts majorly relied upon mental imagery or virtual reality to study the supraspinal control of walking. Recent efforts have been made to develop magnetic resonance imaging (MRI)-compatible devices simulating specific somatosensory and/or motor aspects of walking. However, there exists large variance in the design and functionality of these devices, and as such inconsistent functional MRI (fMRI) observations.

**Methods:**

We have therefore completed a systematic review to summarize current achievements in the development of these MRI-compatible devices and synthesize available imaging results emanating from studies that have utilized these devices.

**Results:**

The device design, study protocol and neuroimaging observations of 26 studies using 13 types of devices were extracted. Three of these devices can provide somatosensory stimuli, eight motor stimuli, and two both types of stimuli. Our review demonstrated that using these devices, fMRI data of brain activation can be successfully obtained when participants remain motionless and experience sensorimotor stimulation during fMRI acquisition. The activation in multiple cortical (e.g., primary sensorimotor cortex) and subcortical (e.g., cerebellum) regions has been each linked to these types of walking-related sensorimotor stimuli.

**Conclusion:**

The observations of these publications suggest the promise of implementing these devices to characterize the supraspinal sensorimotor control of walking. Still, the evidence level of these neuroimaging observations was still low due to small sample size and varied study protocols, which thus needs to be confirmed via studies with more rigorous design.

## Background

The regulation of gait is critical to many activities of everyday life. Aging and age-related conditions (e.g., movement disorders [1], stroke [2], chronic pain [3], etc.) oftentimes diminish gait performance [4, 5] and thus increase the risk of deconditioning, mobility decline, and falls [6, 7] in the older adult population and those suffering from gait disorders (e.g., shuffling gait). Gait is regulated by a complex system that requires ongoing communication between peripheral neuromuscular circuitry [8] and numerous subcortical and cortical networks [9]. In particular, when walking, somatosensory information obtained from mechanoreceptors throughout the body is delivered via peripheral nerves to the central nervous system, where it is processed and integrated with visual and vestibular feedback in numerous cortical and sub-cortical networks and used to develop and execute appropriate motor programs to meet ever-changing environmental and task demands [10]. In addition to the issues in peripheral musculoskeletal systems (e.g., osteoporosis), age and age-related conditions (e.g., stroke and Parkinson’s disease) oftentimes alters the functional characteristics of supraspinal elements (e.g., the brain cortical regions) of walking, resulting in gait disorders. Considerable effort has therefore been placed on the study of the supraspinal control of walking in order to understand the impact of aging and age-related conditions on locomotor control and to identify new targets for preventive and rehabilitative medicine in those suffering from diminished mobility.

Functional magnetic resonance imaging (fMRI) enables the characterization of neural activity with high spatial resolution and can thus provide insight into the supraspinal control of many important functions and behaviors [11]. However, it is challenging to utilize MRI to characterize locomotor control because the individual’s head is required to stay motionless throughout the scan. The majority of efforts to date have thus relied upon mental imagery to study the planning and thought of carrying out movements [12–14], or virtual reality to study the processing of visual feedback related to navigating an environment [15–18]. This research, while important, provides very little information regarding brain-level, somatosensory processing or the sensorimotor control involved in walking [14, 19]. As a result, recent efforts have been made to develop MRI-compatible devices that simulate specific somatosensory and/or motor aspects of walking, while limiting movement of the head. Studies using these devices have indeed provided new insights to locomotor control. At the same time, however, there exists large variance in the design and functionality of these devices, and as such, inconsistent fMRI observations. We have therefore completed a systematic review to (1) summarize the design and functionality of MRI-compatible devices that have been developed to characterize the supraspinal processing of sensorimotor aspects of locomotion, and (2) synthesize available BOLD results emanating from studies that have utilized these devices. The knowledge provided by this work is expected to inform the design of future studies implementing these devices to characterize the supraspinal elements of gait regulation in those suffering from gait disorders.

## Method

### Search strategy

A systematic literature search of PubMed, Web of science, EBSCO MEDLINE, SPORT Discus, Psych-Info, Cochrane library and Scopus was performed with the last search completed on August 27, 2022. The search field focused on the magnetic resonance imaging compatibility of the devices (i.e., MRI-compatible) and the synonyms for gait including lower-extremity motor paradigms that were considered as the clinical surrogates of gait. The following search terms were used to identify relevant literature in the database: (“magnetic resonance imaging compatible” or “MRI compatible”) and (“gait” OR “walk” OR “step*” OR “ambul*” OR “locomot*” OR “lower limb movement” OR “pedal” OR “dorsiflexion” OR “plantarflexion” OR “ankle motion*” OR “foot sole pressure stimul*”). A manual search of the bibliographic references of extracted articles and existing reviews was also conducted to identify studies that were not captured in the electronic searches.

### Inclusion and exclusion criteria

The inclusion criteria were: (1) the device used in the study was MRI-compatible with mechanical structure enabling the control or adjustment of stimulation parameters (e.g., applied force, movement speed of stimulation); (2) the goal of the study or device development was to explore the functional characteristics of the supraspinal regions pertaining to walking-related sensorimotor stimuli. Manuscripts were excluded if they: (1) only contained a descriptive overview of the device design; (2) were systematic reviews, case reports, protocol papers, conference abstracts, or letters to the editor; and (3) were not written in English.

### Data extraction and synthesis

Two authors (YH and JZ) independently performed data extraction, and when disagreement on the extraction of data/information was present, it was discussed with additional authors (DB and BM) until a consensus was achieved. The following data were extracted for each study: (1) device characteristics: simulation protocol of sensorimotor characteristics of gait, design of power supply, execution unit and control unit, materials used to develop the devices, accessories to minimize unwanted or extraneous motions, and the results of MRI compatibility test and the validation test of device performance; and (2) and study characteristics: the information of the study team (e.g., first author, year of publishing, country), participant characteristics, fMRI protocol and design, and the results of head-motion artifacts and fMRI BOLD signal analysis.

## Results

### Study selection

**Fig. 1:**
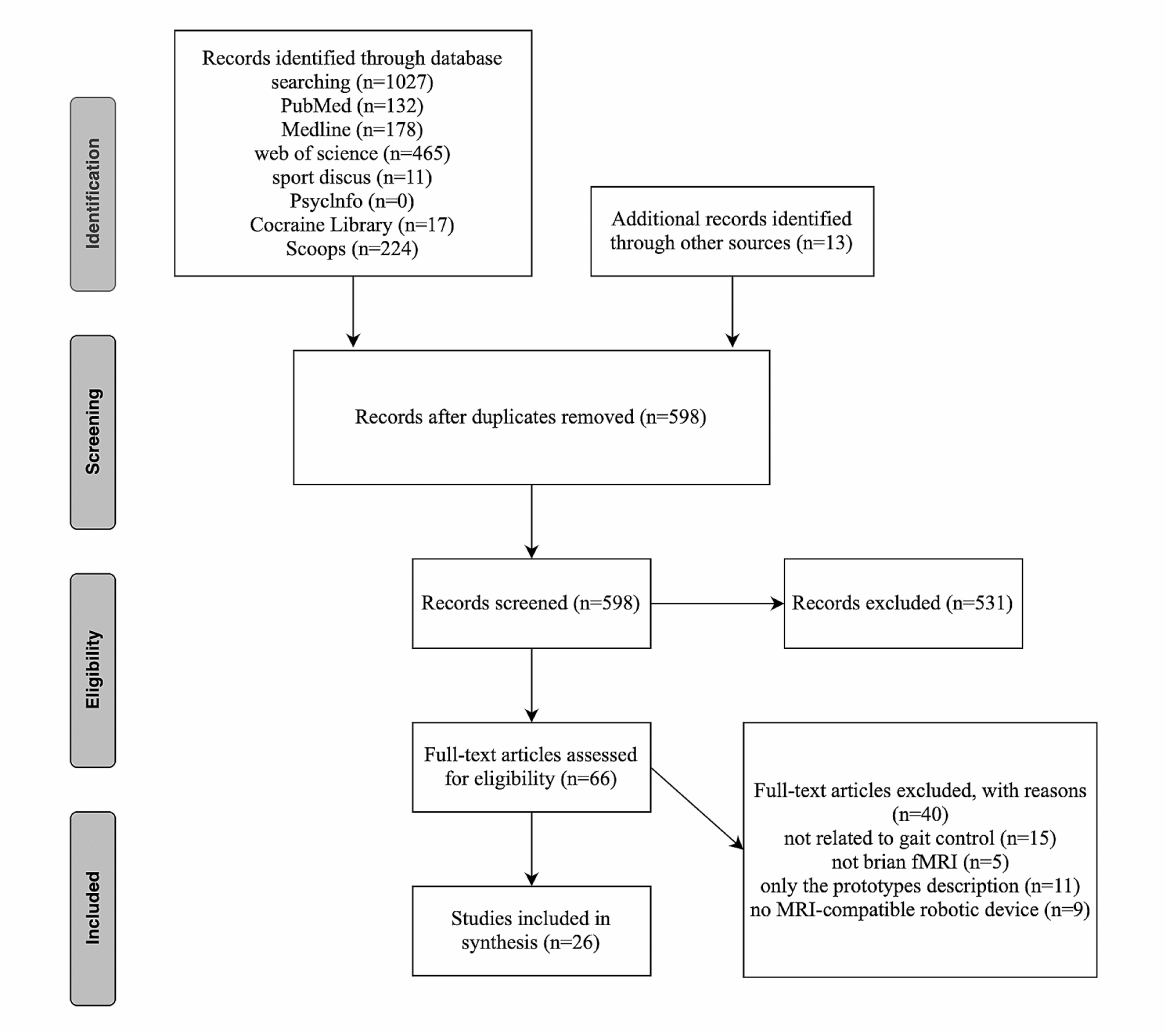
The RISMA flowchart of publication screening. *Abbreviation * PRISMA: Preferred Reporting Items for Systematic Reviews and Meta-Analyses; fMRI: functional magnetic resonance imaging

The data summary and analysis were completed on November 10th, 2022. Figure 1 presents the flow diagram of study selection. Our initial search retrieved 1,027 articles from four databases and 13 additional articles from other sources (e.g., reference lists from original work and review articles). After the removal of duplicates and the screening of title, abstract, and full text for study design and outcomes, 26 original research articles were eligible and included in the systematic review. Reasons for exclusion in this phase included: not related to gait, not an fMRI study, prototype description only, or not an MRI-compatible device with mechanical structure enabling the control or adjustment of stimulation parameters.

### Device feasibility

A total of 13 different devices were reported in 26 total studies, including a foot-sole stimulation system [20], a dual-drive foot-sole stimulator [21], a Korvit boot system [22–24], a foot pedal manipulandum [25], a plantar flexion force measure apparatus [26], a brain discovery pneumatic orthosis (Bra.Di.P.O.) [27, 28], a bipedal device (i.e., one pedal for each of left and right foot) [29], a torque-measuring apparatus [30], a pedaling device [31–33], a pseudogait-magnetic resonance compatible device (pseudogait-MRCD) [34, 35], a cylindrical treadmill device [36], a magnetic resonance compatible stepper (MARCOS) [37–41], and a lower-extremity motion simulator (LOMS) [42–45] (Table 1).

**Table 1:** Device design and functionality

Device		Reference	Type of stimuli	Participant engagement	Accessories to limit motion of head and body	Function	
Foot-sole stimulation system		(Hao et al., 2013) [20]	Foot-sole stimulation	Passive	N.A.	Applies relatively high-pressure stimuli to the foot soles with a programmable waveform and adjustable surface area over which the pressure is applied.	
Dual-drive foot-sole stimulator		(Zhang et al., 2019) [21]	Foot-sole stimulation	Passive	N.A.	Applies controlled dynamic pressure waveform-type stimuli to the foot soles that mimic those experienced when walking.	
The Korvit boot system		(Kremneva et al., 2012) [22] (Labriffe et al., 2017) [23] (Jeanvoine et al., 2022) [24]	Foot-sole stimulation	Passive	Foam blocks	Generates well-controlled, reproducible mechanical stimulation of the plantar surface of the foot by the application of pneumatic pressure on the relevant support zones, in a pattern which reproduces the pressures generated during gait.	
Foot pedal manipulandum		(Trinastic et al., 2010) [25]	Ankle dorsiflexion/ plantarflexion	Active	Foam	Enables twenty degrees of rotation at the ankle, from ten degrees of plantarflexion to ten degrees of dorsiflexion.	
Plantar flexion force measure apparatus		(Noble et al., 2014) [26]	Ankle dorsiflexion/ plantarflexion	Active	A memory foam pillow within the head coil and a Velcro strap around hips and the distal end of legs	Allows participant to produce a plantar flexion exertion against resistance and measure the force of plantar flexion exertions.	
Bra.Di.P.O.	(Belforte et al., 2010) [27] (Belforte et al., 2012) [28]		Ankle dorsiflexion/ plantarflexion	Both passive and active	N.A.	Enables passive foot dorsiflexion and plantarflexion movements, as well as active movements according to a pre-set series of parameters (force, amplitude).	
Bipedal device	(Doolittle et al., 2021) [29]		Lower extremity multi-joint movement	Active	Hook and loop straps to secure the participant’s feet	Enables alternating unilateral and bilateral plantarflexion and dorsiflexion.	
Pedaling device	(Mehta et al., 2009) [31] (Mehta et al., 2012) [32] (Promjunyakul et al., 2015) [33]		Lower extremity multi-joint movement	Active	A beaded vacuum pillow and a “brace” around the head. A chinstrap and additional foam padding was added as needed	Enables forward or backward pedaling against a frictional load with rates up to 80 RPM.	
Torque-measuring apparatus	(Newton et al., 2008) [30]		Lower extremity multi-joint movement	Active	N.A.	Enables to measure torques generated at the ankle, knee and hip joints simultaneously by using 6-axis non-magnetic load cell, as well as providing visual feedback of the torque at the relevant joint to the subject.	
Pseudogait-MRCD	(Martínez et al., 2014) [34] (Martínez et al., 2016) [35]		Lower extremity multi-joint movement	Active	A vacuum pillow (Siemens Cushion Head 4,765,454) and elastic Velcro straps placed over the hips and thighs	Allows participant to perform voluntary step-like movements and avoid hip movements while lying in the supine position.	
Cylindrical treadmill device		(Toyomura et al., 2018) [36]	Lower extremity multi-joint movement	Active	Several belts and small cushions inserted into the space between the headset and head coil	Allows participant to turn the cylindrical treadmill from the outside to perform stepping movements.	
MARCOS		(Hollnagel et al., 2011) [37] (Hollnagel et al., 2013) [38] (Jaeger et al., 2014) [39] (Jaeger et al., 2015) [40] (Jaeger et al., 2016) [41]	Lower extremity multi-joint movement and extra foot-sole stimulation	Both passive and active	Shoulder belts, a vacuum pillow placed at the back, a rigid hip fixation, and a head bowl in combination with the Crania fixation	Enables programmable, highly repetitive periodic active or passive leg movements comprised of hip, knee, and ankle joint displacements as well as GRF applied to foot soles.	
LOMS		(Takahiro et al., 2011) [42] (Ikeda et al., 2012) [43] (Takahiro et al., 2013) [44] (Ikeda et al., 2015) [45]	Lower extremity multi-joint movement and extra foot-sole stimulation	Both passive and active	N.A.	Enables to control joint angle trajectory and joint togue at each joint independently enables various active and passive movements of the user’s lower extremities and has foot stimulating parts at its two soles to simulate floor reactive force.	

*Abbreviation* Bra.Di.P.O., brain discovery pneumatic orthosis; RPM, Revolutions Per Minute; pseudogait-MRCD, pseudogait-magnetic resonance compatible device; MARCOS, magnetic resonance compatible stepper; LOMS, lower-extremity motion simulator; GRF, Ground Reaction Force

### Device design

**Fig. 2:**
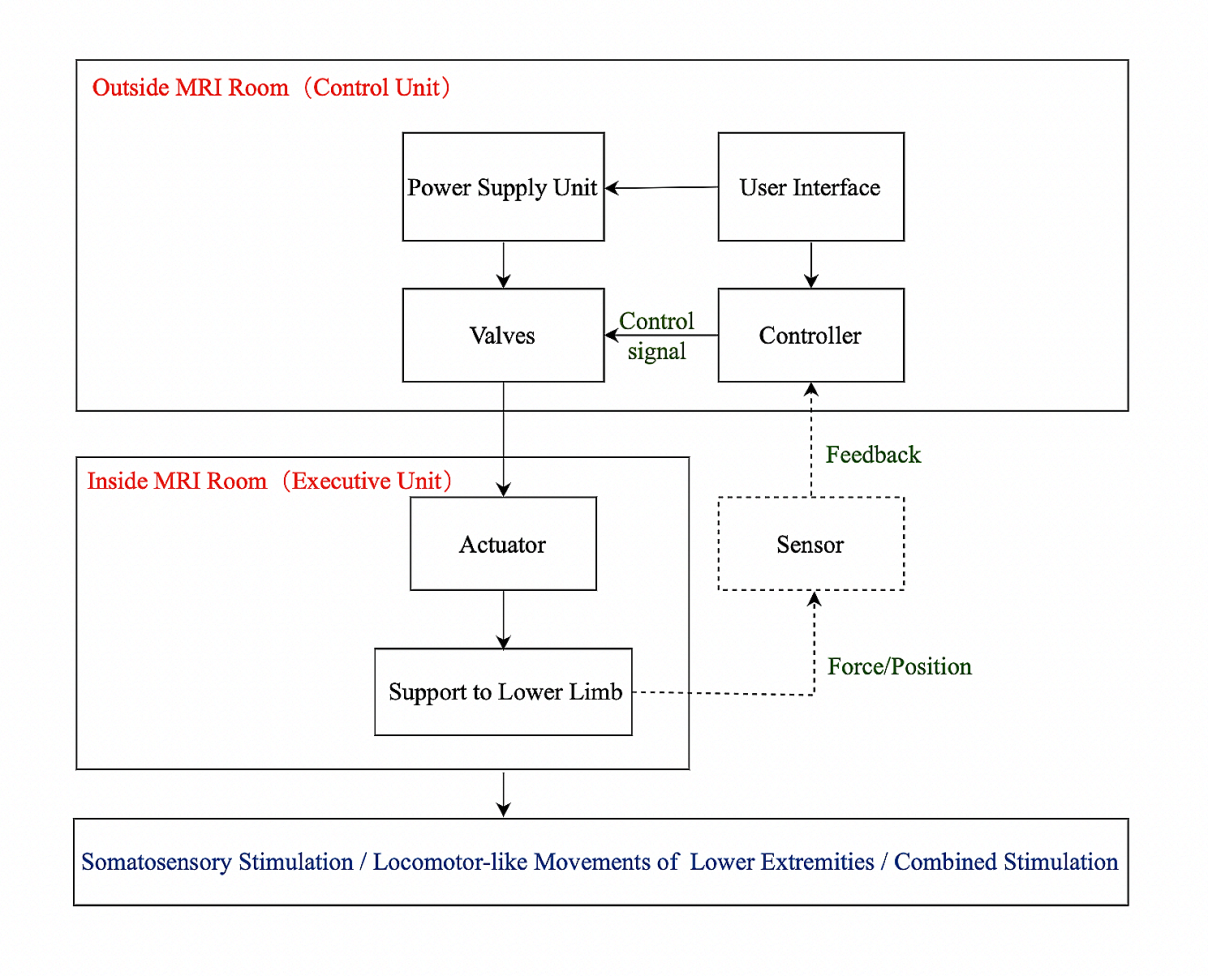
The conceptual diagram of device structure

The structure of included devices can be divided into three main parts: the power supply unit, the execution unit that is oftentimes placed in the MRI scan room, and the control unit placed outside the scan room (Fig. 2; Table 2).

**Table 2:** Information on the components of device

Device	Reference	Power supply unit	Execution unit			Control unit				
			Support to the lower limb	Actuator	Sensor	Controller	Valve	Data acquisition system	User interface	
Foot-sole stimulation system	(Hao et al., 2013) [20]	An air compressor	A plastic boot	A linear pneumatic actuator consisting of a single-acting, single rod air cylinder (CG1BN32-40-XC6, SMC, Tokyo, Japan)	N.A.	A microprocessor (MSP 430 F168, Texas Instruments, Dallas, TX, USA)	A proportional electro-pneumatic valve, five-port air-operated valve	N.A.	An LCD display and four control buttons	
Dual-drive foot-sole stimulator	(Zhang et al., 2019) [21]	An air-compressor (GL0205, Greeloy, Shanghai, China)	A plastic boot	Two air cylinders (CG1BN32-40XC6, SMC, Tokyo, Japan)	N.A.	A microcontroller (MSP430F168, Texas Instruments, Dallas, TX, United States)	Two five-port solenoid valves (SY51205LZD-C6, SMC Corporation, Tokyo, Japan), a proportional valve (ITV2030-312 L, SMC Corporation, Spain)	N.A.	A custom-developed user interface based on Matlab (The mathworks, Inc., Natick, MA, United States)	
The Korvit boot system	(Kremneva et al., 2012) [22] (Labriffe et al., 2017) [23] (Jeanvoine et al., 2022) [24]	A compressor	A plastic boot	Pneumatic ortheses (with cylinder pneumatic cameras built in the insoles)	N.A.	N.A.	N.A.	N.A.	N.A.	
Foot pedal manipulandum	(Trinastic et al., 2010) [25]	N.A.	A customized pedal	N.A.	N.A.	N.A.	N.A.	N.A.	N.A.	
Plantar flexion force measure apparatus	(Noble et al., 2014) [26]	N.A.	syringes	N.A.	A pressure transducer	N.A.	N.A.	N.A.	N.A.	
Bra.Di.P.O.	(Belforte et al., 2010) [27] (Belforte et al., 2012) [28]	N.A.	A customized pedal	A pneumatic actuator	A custom-built analogue optical encoder and a pressure transducer	N.A.	5 eletrovalves, 3 OR valves, 2 flow regulators, a pressure reducer, a pneumatically controlled bistable valve, a low-high pressure valve, a plunger valve	N.A.	N.A.	
Bipedal device	(Doolittle et al., 2021) [29]	N.A.	A customized pedal	N.A.	Reaction torque sensors (Transducer Techniques, TRS2K); a glass/ceramic hybrid electrolytic sensor (angle sensor)	N.A.	N.A.	A data acquisition device (Quanser, Q8-USB) via Simulink (mathworks)	N.A.	
Pedaling device	(Mehta et al., 2009) [31] (Mehta et al., 2012) [32] (Promjunyakul et al., 2015) [33]	N.A.	A customized pedal	N.A.	An optical encoder (model TD 5207, Micronor Inc., CA)	N.A.	N.A.	Data acquisition software (micro 1401 mk II and Spike, Cambridge Electronics Design, UK)	A laptop computer	
Torque-measuring apparatus	(Newton et al., 2008) [30]	N.A.	A customized pedal	N.A.	A 6-axis load cell (model 45E15A; Woodland, CA)	N.A.	N.A.	A 12-bit data acquisition card (DAS1200, Measurement Computing, Middleboro, MA)	Custom software (Matlab, Mathworks, Natick MA)	
Pseudogait-MRCD	(Martínez et al., 2014) [34] (Martínez et al., 2016) [35^]^	N.A.	A special treadmill	N.A.	Four cable potentiometers, optical encoder (Hewlett Packard HEDS-5540, with 10-bits resolution)	N.A.	N.A.	Device Acquisition System (a data acquisition PCI card (Sensoray Model 626)	A personal computer	
Cylindrical treadmill device	(Toyomura et al., 2018) [36]	N.A.	A special treadmill	Pneumatic actuation	N.A.	N.A.	N.A.	N.A.	N.A.	
MARCOS	(Hollnagel et al., 2011) [37] (Hollnagel et al., 2013) [38] (Jaeger et al., 2014) [39] (Jaeger et al., 2015) [40] (Jaeger et al., 2016) [41]	N.A.	A customized lower limb exoskeleton	Two pneumatic actuations (DNC 40-320-P-K10-S11 (knee), DNC 32-350-P-K10-S11 (foot) Festo, Esslingen am Neckar, Germany)	Optical encoders (Type MS20, RSF Elektronik AG, Schwerzenbach, Switzerland) with a ceramic scale and one foil potentiometer (MTP L22, Resenso, Ins, Switzerland) and resistive strain gauges (force sensors)	A low-level pneumatics controller and a high-level stepping controller	Proportional flow valves, pressure-control valves, a proportional multiway valve	Data acquisition boards	Two personal computers	
LOMS	(Takahiro et al., 2011) [42] (Ikeda et al., 2012) [43] (Takahiro et al., 2013) [44] (Ikeda et al., 2015) [45]	An air compressor	A customized lower limb exoskeleton	Two Mckibben-type pneumatic artificial muscles	Six rotary potentiometers (Murata Manufacturing Co., Ltd.)	A controller(SH4: General Robotix, Ibaraki, Japan)	Two solenoid valves	N.A.	N.A.	

*Abbreviation* Bra.Di.P.O., brain discovery pneumatic orthosis; pseudogait-MRCD, pseudogait-magnetic resonance compatible device; MARCOS, magnetic resonance compatible stepper; LOMS, lower-extremity motion simulator; LCD, liquid crystal display

For power supply, four devices used an air compressor to drive a pneumatic actuator [20–24, 42–45], while such information is missing for the other nine.

The execution units consist of three structures: support to the lower limb, sensor, and actuator. Types of lower limb support included a plastic boot (number of devices (N) = 3) [20–24], syringes (*N* = 1) [26], a customized pedal (*N* = 5) [25, 27–33], a special treadmill (*N* = 2) [34–36], and a customized lower limb exoskeleton (*N* = 2) [37–45]. Sensor types included force/torque sensors (*N* = 2) [26, 30], displacement sensors (optical encoders [31–35] or a rotary potentiometer [42–45])(*N* = 3), and the combination of at least two types of sensors (e.g., the Bra.Di.P.O. used both a custom-built analogue optical encoder and a pressure transducer [27, 28])(*N* = 3) [27–29, 37–40]. Five devices did not report sensor information. For the types of actuators, pneumatic actuators were used in seven devices [20–23, 27, 28, 36–45], while such information was missing for the other six.

Control units consisted of valves, a controller, a data acquisition system, and a user interface. The details of valves were provided in five devices [20, 21, 27, 28, 37, 38, 42–44], and that of controller in four [20, 21, 37, 42, 44], data acquisition system in five [29–32, 34, 37], and user interface in six devices [20, 21, 30–32, 34, 37] (See Table 2 for details).

In order to prevent unwanted task-related movement of the body as well as the extraneous motion of head that may interfere with image quality, all devices implemented different accessories, such as Velcro straps or belts (*N* = 6) [26, 29, 31–39, 41], triangular platforms (*N* = 4) [26, 30, 34, 35], cushion (*N* = 1) [36], and/or support in the head coil of MRI scanner to help secure the head (i.e., pillows (*N* = 4) [26, 31–35, 37–41] or foam blocks (*N* = 3) [23–25, 31–33].

### Device compatibility with fMRI

MRI compatibility of seven out of the thirteen devices were tested via phantom-based imaging quality tests and reported in nine publications [20, 21, 31, 34, 36, 37, 42, 43, 45]. Uniquely, the compatibility of one device was also tested when human participants were performing tasks (e.g., voluntary motion of the lower extremities with and without wearing the lower-extremity motion simulator ) [42, 43]. Specifically, two studies measured spatial signal-to-noise ratio (SNR) [31, 45]; two measured temporal SNR [36, 37]; one measured both spatial SNR and temporal SNR [34]; one measured spatial SNR, the signal-to-fluctuation-noise ratio (SFNR) and the field map mean [20]; one measured spatial SNR, SFNR and the field non-uniformities [21]; and the other two measured the signal intensity and dispersion [42, 43] (Table 3). Additionally, both non-metallic materials including plastic, rubber, and polyvinyl chloride (PVC) and non-magnetic metal including aluminum, bronze, brass, and stainless steel were used to manufacture devices to ensure the MRI compatibility (Table 3).

**Table 3:** Information on the compatibility of device

Device	References	Materials	Compatibility test	Device performance verification	Head-motion artifacts (mean)
Foot-sole stimulation system	(Hao et al., 2013) [20]	Aluminum, nonferromagnetic plastic and nylon	Phantom-based image quality test (three conditions: powered on, powered off, absent from room; index: anatomical SNR/functional SFNR/field map mean).	Comparison of preprogrammed foot stimulation with the actual stimulation applied to the foot soles.	< 1 mm; < 1 °
Dual-drive foot-sole stimulator	(Zhang et al., 2019) [21]	Plastic, aluminum and nylon	Phantom-based image quality test (three conditions: powered on, powered off, absent from room; index: anatomical SNR/functional SFNR/field non-uniformities).	Similarity between real foot sole pressures experienced when walking and those simulated by the stimulation system.	< 1 mm
The Korvit boot system	(Kremneva et al., 2012) [22]	Plastic, rubber	N.A.	N.A.	N.A.
	(Labriffe et al., 2017) [23]		N.A.	N.A.	One participant was excluded
	(Jeanvoine et al., 2022) [24]		N.A.	N.A.	Two participants were excluded
Foot pedal manipulandum	(Trinastic et al., 2010) [25]	N.A.	N.A.	N.A.	N.A.
Plantar flexion force measure apparatus	(Noble et al., 2014) [26]	PVC	N.A.	N.A.	< 3 mm
Bra.Di.P.O.	(Belforte et al., 2010) [27]	Aluminum, bronze, brass, polymer and Derlin	N.A.	N.A.	N.A.
	(Belforte et al., 2012) [28]		N.A.	Dynamic performance of the control circuit (e.g., comparison of experimental and theoretical results)	N.A.
Bipedal device	(Doolittle et al., 2021) [29]	A mixture of aluminum; various types of stainless steel; plastic; rubber; glass/ceramic and fluid	N.A.	Test-retest reliability of measurements (i.e., angle displacement, head motion, task-related BOLD signal during left and right foot movement)	0.10 ± 0.02 mm
Pedaling device	(Mehta et al., 2009) [31]	PVC, Delrin, phenolic, nylon and wood	Phantom-based image quality test (four conditions: Phantom, Phantom + bike, Phantom + bike + electronics, Phantom + bike + electronics + movement; index: the percent change in brightness and spatial SNR).	N.A.	1 mm
	(Mehta et al., 2012) [32]		N.A.	N.A.	< 0.5 mm; < 0.5°
	(Promjunyakul et al., 2015) [33]		N.A.	N.A.	< 3 mm
Torque-measuring apparatus	(Newton et al., 2008) [30]	Fiberglass, aluminum, brass, polyethylene	N.A.	Reliability of motor performance (e.g., the mean amplitude of the torques) and fMRI-derived measures of brain activity across two time points in each participant	N.A.
Pseudogait-MRCD	(Martinez et al., 2014) [34]	High molecular weight polyethylene, nonmagnetic mental and plastic	Phantom-based image quality test (four conditions: phantom alone; with the device; with the device and movement; phantom alone; index: the differences in the mean images; percent change in brightness; spatial SNR and temporal SNR)	N.A.	< 3 mm
	(Martínez et al., 2016) [35]		N.A.	N.A.	< 2 mm
Cylindrical treadmill device	(Toyomura et al., 2018) [36]	Wood, brass, rubber and methacrylate	Phantom-based image quality test (four conditions: no device, with device; with rotation; no device; index: temporal SNR)	N.A.	< 3 mm
MARCOS	(Hollnagel et al., 2011) [37]	PVC, aluminum and brass	Phantom-based image quality test (three conditions: no device (1st baseline), cables connected, moving device, and no device (2nd baseline); index: temporal SNR).	N.A.	< 2 mm
	(Hollnagel et al., 2013) [38]		N.A.	Benefit of the ILC in the control performance (e.g., the shift/difference between the maximum phase of the expected and measured trajectories of position or force).	< 2.5 mm
	(Jaeger et al., 2014) [39]		N.A.	N.A.	N.A.
	(Jaeger et al., 2015) [40]		N.A.	Test-retest reliability of motor performance (i.e., knee amplitude, stepping frequency and foot force) and brain activation of the robot-aided experimental fMRI paradigm (i.e., intra-class ICC computed from the single-subject and group activation maps for five ROIs).	Eight participants were excluded
	(Jaeger et al., 2016) [41]		N.A.	Performance of the robot (i.e., the variability of the delivered foot load; the congruence of the knee amplitude and stepping frequency across load levels).	N.A.
LOMS	(Takahiro et al., 2011) [42]	ABS, acrylic and nonmagnetic stainless steel	Phantom-based image quality test (e.g., signal intensity of fMRI scanning related to the distance between LOMS and head coil), functional imaging assessment (moved the lower extremities voluntarily with or without wearing LOMS) and motion capturing assessment (the angles of the hip joints in three cases).	N.A.	N.A.
	(Ikeda et al., 2012) [43]		Phantom-based image quality test (e.g., signal intensity of fMRI scanning related to the distance between LOMS and head coil), functional imaging assessment (moved the lower extremities voluntarily with or without wearing LOMS) and motion capturing assessment (the angles of the hip joints in three cases).	N.A.	N.A.
	(Takahiro et al., 2013) [44]		N.A.	N.A.	N.A.
	(Ikeda et al., 2015) [45]		Phantom-based image quality test (three tests: different distance/in or out of room/movement or not; index: spatial SNR).	N.A.	N.A.

*Abbreviation* Bra.Di.P.O., brain discovery pneumatic orthosis; RPM, Revolutions Per Minute; pseudogait-MRCD, pseudogait-magnetic resonance compatible device; MARCOS, magnetic resonance compatible stepper; LOMS, lower-extremity motion simulator; SNR, signal-to-noise ratio; SFNR, signal-to-fluctuation-noise; ICC, intraclass correlation coefficient; ROI, region of interest; PVC, polyvinyl chloride; ABC, Acrylonitrile butadiene styrene

### Device functionality

The overarching goal of included devices was to simulate walking-related somatosensory and/or motor experiences of walking over ground, during the MRI scan, thereby enabling study of related brain activity. As such, devices were designed to apply somatosensory stimuli to the foot soles (*N* = 3), induce locomotor-like movements of the lower extremities (including single-joint movement of ankle (*N* = 4), and multi-joint movements (*N* = 4)), or induce locomotor-like movements of the lower extremities while simultaneously applying somatosensory stimuli to the foot soles (*N* = 2). Additionally, three devices were designed for passive participant engagement, seven for active engagement to initiate movements by participants, and the other three for both functionalities **(**Table 1**).**

Three devices were designed to apply somatosensory stimuli to the soles of one or both feet [20–24] that mimic the ground reaction forces (GRF) experienced when walking. Specifically, the foot-sole stimulation system was designed to apply a sinusoidal pressure waveform with a peak force of 200 N and in a range of frequency from 1 to 10 Hz to one region of one foot sole [20]. Two other similar devices were designed to apply pressure stimuli to multiple areas of one or both soles: the dual-drive foot-sole stimulator was designed to apply programmable waveform-type stimuli reproducing the pressure waveforms of each participant as recorded during walking over an instrumented pressure insole prior to the scan [21]; the Korvit boot system was designed to apply two different forms of stimulation including “gait-like” and “chaotic” sequences [22–24].

Eight devices were designed to enable locomotor-like movements of the lower extremities. Five of these devices were designed to induce single-joint movement (i.e., ankle dorsiflexion and plantarflexion) and three were designed to induce multi-joint movements. Of the five single-joint devices, two were designed to enable unilateral movement [25, 30]. The other three were created to enable either unilateral or bilateral ankle dorsiflexion and plantarflexion with unique functions: one device provided real-time feedback of plantar flexion exertion in order to have participants attempt to achieve target forces [26]; one device was created to enable synchronization of the angle and torque characteristics of ankle movements in order to ensure consistency of task performance across trials [29]; and a final device (i.e., Bra.Di.P.O) was created to induce passive ankle motion patterns [27, 28]. Of the three devices enabling multi-joint movements, the pedal device was created to induce movements at different speeds without the restriction of knee motion [31–33]; the cylindrical treadmill device was created to enable participants to perform stepping movements by moving their legs to rotate a cylinder [36]; and the pseudogait-MRCD consisted of a vertical treadmill connected to a triangular platform that was create to enable participants to perform voluntary step-like movements [34, 35].

The final two devices were designed to enable locomotor-like movements of the lower extremities combined with extra somatosensory stimuli [26–34]. Specifically, the MARCOS device was described as being actuated by two pneumatic cylinders: one attached to knee orthoses allowing predefined flexion and extension movements of each leg in the sagittal plane, and the other attached to the shoe rendering external loads of up to 400 N along the cranio-caudal body axis to the foot-sole [37–41]. The LOMS device was created to enable three degree-of-freedom motion of each leg and simultaneously applies the ground reaction force of walking to foot soles via the foot stimulating parts [42–45].

### Validation of device performance

Device performance was assessed in eight devices **(**Table 3**)**. The similarity between the pressure as experienced during over ground walking and those simulated by the devices was tested for one device [21]; theoretical outputs (e.g., the output calculated from the formula using preprogrammed parameters) and the results obtained from the actual tests were compared in two devices [20, 28]; the performance of iterative learning in the controller for one (i.e., the shift/difference between the maximum phase of the expected and measured trajectories of position or force) [38]; and the reliability of motion (e.g., the mean amplitude of the torques) across different time points [29, 30, 40] and/or load levels [41] was tested for three. No such information was provided for the other five devices [22–27, 31–36, 39, 42–45].

### The fMRI studies

#### Study characteristics

The 26 included fMRI studies were completed by 12 groups from nine countries. A total of 371 participants were included across studies. The sample size in the included studies ranged from one to 67. The sex of participants was reported in 17 of 26 studies. Twenty-five studies focused on healthy participants and one study focused on participants with brain damage due to stroke [33]. Ten studies reported both the age range and average age (one of which was median rather than mean [30]) of participants, five reported only the age range, seven reported only the average age (one of which had only one participant [37]), and four did not report age information (Table 4).

**Table 4:** The design of fMRI task and key findings

Studies	Country	Study population	fMRI test	Task blocks	Key findings for brain activation
(Hao et al., 2013) [20]	China	7 healthy participants aged 23–27(4 M/3F)	Supraspinal activation in response to foot sole stimulation.	Three repetitions of 30s stimulation applied to a circular area 4 cm of the right foot sole.	Significant activation contralaterally within the S1, S2 and M1, and bilaterally within the S2 during single-point sinusoidal stimulation were observed.
(Zhang et al., 2019) [21]	China	9 healthy participants aged 20–29	Supraspinal activation in response to foot sole stimulation.	A block-designed 3.5-min stimulation protocol consisting of alternating blocks of 30 s-Rest and 30s-Stim.	Significant activation within the SMA, supramarginal gyrus, paracingulate gyri, INS, precentral gyrus, middle temporal gyrus, and hippocampus during stimulation that mimic walking were observed.
(Kremneva et al., 2012) [22]	Russia	12 healthy participants aged 22–42(6 M/6F; A = 28.8)	Supraspinal activation during mechanical stimulation of plantar support zones in different modes (i.e., standing simulation and slow walking simulation modes).	Two tasks (simulation of standing or slow walking). Alternating rest and stimulation for 3 times, for a total of 3 min 53s each task.	Significant activation in the S1, PMC, dlPFC and INS during mechanical stimulation of the plantar support zones were observed. The involvement of the PFC during simulation of standing and a broad involvement of the S1 and S2 during simulation of slow walking were found.
(Labriffe et al., 2017) [23]	France	18 healthy participants aged 20–40(11 M/7F; A = 27 ± 4.7)	Supraspinal activation under three conditions: gait imagination task, organized (“gait like”) and chaotic sequences of plantar stimulations.	Three sessions, each with two consecutive conditions (rest and stimulation). Each condition was performed for 19s and repeated nine times, for a total session duration of 5 min 41s.	Mechanical plantar stimulation activated M1 and S2 bilaterally. The common patterns of activation between mental imagery and gait-like plantar stimulation were observed, specifically in SMA-proper bilaterally and right pre-SMA. There was no difference between the organized and chaotic patterns of stimulation.
(Jeanvoine et al., 2022) [24]	France	67 healthy participants aged 20–77(32 M/35F; A = 49.2 ± 18.0)	Supraspinal activation under two conditions: organized (“gait like”) and chaotic sequences of plantar stimulations.	Two sessions, each with two consecutive conditions (rest and stimulation). Each condition was performed for 19s and repeated nine times, for a total session duration of 5 min and 42s.	Brain areas (pre-SMA, mid-DLFPC,V1) involved in age-related changes in somatosensory processing of gait.
(Trinastic et al., 2010) [25]	USA	8 intact adults aged 25–57 (6 M/2F; A = 31.5)	The difference in supraspinal activation between active ankle dorsiflexion and plantarflexion.	Fifteen sets of stimuli, each with two active ankle dorsiflexion and two active plantarflexion, for a duration of 288.1s.	Ankle dorsiflexion and plantarflexion may be controlled by both shared and independent neural circuitry.
(Noble et al., 2014) [26]	British	11 participants with normal vision aged 19–34(4 M /7F)	Supraspinal activation under five conditions: R15-ONLY; L15-ONLY; BILAT15; R30-ONLY; L30-ONLY.	Four conditions in random order with 10-16s rest, each condition plantar flexion 3 times for 5s.	Greater levels of activation during bilateral exertions may arise from interhemispheric inhibition, as well as from the greater need for motor coordination and visual processing.
(Belforte et al., 2010) [27]	Italy	one healthy participant	Supraspinal activation in response to active and passive foot movement.	Alternating plantarflexion/dorsiflexion and rest for 12s each, for a total of 6.6 min.	Both active and passive movement induced activation in S1/M1 and SMA, and active movements uniquely induced activation in thalamic, frontal and cingulated regions, while passive movements induced activation in temporal and parietal areas.
(Belforte et al., 2012) [28]	Italy	around 10 healthy participants	Supraspinal activation in response to active and passive foot movement.	Alternating plantarflexion/dorsiflexion and rest for 12s each, for a total of 6.6 min.	Both active and passive movement induced activation in S1/M1 and SMA, and active movements uniquely induced activation in thalamic, frontal and cingulated regions, while passive movements induced activation in temporal and parietal areas.
(Doolittle et al., 2021) [29]	USA	20 healthy participants aged 21–37 (12 M/8F; A = 26)	Supraspinal activation during unipedal and bipedal movement.	Two repetitions of four blocks of dorsiflexion-plantarflexion (right only, left only, both, and rest), for 20s each.	No significant difference in the BOLD signal between unipedal and bipedal motion in the ROI explored were observed.
(Mehta et al., 2009) [31]	USA	10 healthy participants aged 21–53 (6 M/4F; A = 31)	Supraspinal activation in response to pedaling at a rate of 30 RPM.	A single run consisted of 30s of pedaling and 30s of rest alternated 4 times.	Consistent with previous literature, the medial S1 and M1, PMA, SMA and Cb are involved in pedaling.
(Mehta et al., 2012) [32]	USA	10 healthy participants aged 21–53 (6 M/4F; A = 31)	Supraspinal activation during slow (30 RPM), fast (60 RPM), passive (30 RPM), and variable rate pedaling.	A block design consisted of 3 runs. Each run consisted of 4 repetitions of pedaling for 30s and resting for 30s.	Significant activity in M1, S1, SMA, and Cb during pedaling that increased with increasing pedaling rate and complexity. Similar levels of cortical and Cb activity were present during active and passive pedaling.
(Promjunyakul et al., 2015) [33]	USA	14 stroke (5 M/9F; M = 54.5 ± 12.3) and 12 control participants (6 M/6F; A = 53.4 ± 13.1)	Supraspinal activation in response to pedaling in individuals with stroke and age-matched controls.	A single run consisted of 30s of pedaling and 30s of rest alternated 4 times.	Brain activation volume in BA6 and Cb during pedaling was reduced in people post-stroke, as compared to age-matched controls.
(Newton et al., 2008) [30]	USA	9 healthy participants aged 21–58 (4 M/5F; Median = 39)	Supraspinal activation of three isolated lower limb isometric contractions: ankle dorsiflexion, ankle plantarflexion and knee extension.	Four blocks of cued contractions (each 32s in length), interleaved with five rest periods of 28s duration.	Significant BOLD signal increases were observed in L.SM1 in the paracentral lobule and in M2 for ankle dorsiflexion, ankle plantarflexion and knee extension. Within these areas there was substantial overlap of the motor representations though differential activation was observed in SM1, with greater activation of inferior paracentral lobule during knee extension than for either ankle task.
(Martínez et al., 2014) [34]	Spain	19 participants (10 M/9F; A = 33 ± 5)	Supraspinal activation in response to stepping while selecting subject’s individual comfortable amplitude.	Twelve repetitions of 10-s blocks of voluntary alternating strides of the lower limbs and 30-s blocks of rest with a total duration of 8 min.	Stepping generates extensive bilateral activations in several cortical and subcortical brain regions know to be related to motor execution and motor control.
(Martínez et al., 2016) [35]	Spain	19 healthy participants aged 25–42 (10 M/9F; A = 33.29 ± 5.8)	Supraspinal activation in response to stepping at different paces (0.8, 1.2 or 1.75 steps per second).	Two runs, each run consisted of 18 motor blocks (six repetitions by condition) of 10s duration and the corresponding resting of 30s with a total duration of 12 min.	Brain activity patterns showed similar BOLD responses across pace conditions though significant differences were observed in parietal and cerebellar regions.
(Toyomura et al., 2018) [36]	Japan	20 healthy participants aged 20–23 (14 M/6F; A = 21.9)	Supraspinal activation in response to lower-limb movement in three speed conditions (slow, medium, fast).	Brain activity patterns showed similar BOLD responses across pace conditions though significant differences were observed in parietal and cerebellar regions.	The post/pre-central gyrus and Cb showed significant activity during the movements.
(Hollnagel et al., 2011) [37]	Switzerland	one participant aged 30 (M)	Supraspinal activation of the single subject during all nine tasks (e.g., alternating passive stepping at 0.5 Hz).	Nine movement paradigm, each executed 30 times for 10s (five steps for 0.5 Hz), interleaved by 5s rest.	The more “uncommon” the motor task and the more “active” and “challenged” the subject was, the more activity was elicited within the sensorimotor brain areas.
(Hollnagel et al., 2013) [38]	Switzerland	13 healthy participants aged 22–32 (3 F/10 M)	Supraspinal activation during each training mode (active, passive and assist-as-needed stepping).	Three modes, each with 9 trails. Each trial consisted of 30s moving followed by 10s rest.	Active stepping elicited significant activation in an extensive sensorimotor network including medial M1 and L.PMC as well as activation in the Vermis and R.Cb. Passive stepping elicited activation in medial M1 and PMA in the left cerebral hemisphere but not the Cb. Stepping with assist-as-needed led to significant activations in the L.SM1 and bilaterally in the superior parietal lobe.
(Jaeger et al., 2014) [39]	Switzerland	24 healthy right-handed and-footed participants (16 M/8F; A = 27 ± 4)	Supraspinal activation in response to active and passive, bilateral, periodic, multi-joint, lower limb motor control.	Two runs, each with 15 trials of movement, each trial duration was 10s.	Active and passive stepping engaged several cortical and subcortical areas of the sensorimotor network, with higher relative activation of those areas during active movement.
(Jaeger et al., 2015) [40]	Switzerland	24 healthy right-handed and-footed participants (16 M/8F; A = 27 ± 4)	Supraspinal activation in response to repeated active and passive stepping movements.	Two runs, each with 15 blocks of movement, interleaved with 15 blocks of a control condition. Each block lasted 10s and was followed by 9.075s of image acquisition.	Activations during passive movements are less robust over repeated measurement sessions than those during active movements despite lower variability of motor performance during passive movements.
(Jaeger et al., 2016) [41]	Switzerland	16 healthy participants	Supraspinal activation during active and passive stepping across simulated ground reaction forces (0, 20, and 40% of individual body weight).	A block design consisted of 6 runs in random order. Each run consisted of 15 blocks of movement, and 15 blocks of a control condition. Each block lasted 10s and was followed by 9.075s of image acquisition.	A significant modulation of brain activation in sensorimotor areas by the load level could neither be demonstrated during active nor during passive stepping.
(Takahiro et al., 2011) [42]	Japan	one participant	Supraspinal activation during the subject moved the lower extremities voluntarily with or without wearing LOMS.	Four repetitions of 25s rest and 25s gait-like motion.	Activations in the M1, SMA and Cb during flexion and extension of lower extremities were observed.
(Ikeda et al., 2012) [43]	Japan	10 healthy participants aged 20	Supraspinal activation in response to gait-like movement with or without floor reactive force.	Eight repetitions of 30s rest and 36s task.	Activation with floor reactive force at the motor cortex was different from the activation without floor reactive force and, any region of the brain proper to floor reactive force was observed.
(Takahiro et al., 2013) [44]	Japan	one health participant aged 20	Supraspinal activation during active and passive gait-like movement simulated by LOMS.	Four repetitions of 30s rest and 36s motion.	Brain activation area in sensory area during passive gait-like motion was broader than one during active gait-like motion.
(Ikeda et al., 2015) [45]	Japan	13 healthy participants aged 20	Supraspinal activation in response to gait-like motion.	Four repetitions of 25s rest and 25s gait-like motion.	Activated regions are the medial M1 and the medial S1 which are related to gait motion.

*Abbreviation*Bra.Di.P.O., brain discovery pneumatic orthosis; RPM, Revolutions Per Minute; pseudogait-MRCD, pseudogait-magnetic resonance compatible device; MARCOS, magnetic resonance compatible stepper; LOMS, lower-extremity motion simulator; M, man; F, female; A, average; R15-ONLY, right foot at 15% of maximal voluntary contraction (MVC); L15-ONLY, left foot at 15% of MVC; BILAT15, with both feet simultaneously with each foot at 15% MVC; R30-ONLY, right foot at 30% of MVC; L30-ONLY, left foot at 30% of MVC; RPM, Revolutions Per Minute; A, anterior; L, left; R, right; S1, primary somatosensory cortex; M1, primary motor cortex; S2, secondary somatosensory cortex; SMA, supplementary motor area; INS, insula; PMC, premotor cortex; dlPFC, dorsolateral and prefrontal cortices; V1, primary visual areas; PCUN, precuneus; Cb, cerebellum; ROI, regions of interest; BA6, Brodmann’s area 6; BOLD, blood oxygen level dependent; SM1, primary sensorimotor cortex; M2, secondary motor cortex

Only two of the 26 studies reported on safety testing. One reported that the safety of their device was examined in a previous iterative pilot testing, but did not report details of the safety test [29], and the other reported that it was confirmed that all components of the device were not attracted by a U-shaped neodymium magnet [45].

Fifteen of 26 studies reported information related to head-motion artifacts associated with usage of the devices [22, 25, 27, 28, 30, 39, 41–45]. Twelve of these studies reported that head motion was less than 3 mm [20, 21, 26, 29, 31–38]; the other three did not report the details of head motion, but stated that the data with excessive head motion were excluded from analyses [23, 24, 40] (Table 3).

Twenty-three of 26 studies utilized a block-design fMRI paradigm and the remaining three studies utilized event-related designs [25, 26, 37]. Eighteen of 26 studies reported the details of imaging processing procedure [20–26, 28–36, 39, 40], while such information is missing for the other eight. Specially, four of the 18 studies reported the utilization of the sparse sampling imaging protocol [31–33, 39].

For the comparisons performed in the included studies, 17 of 26 studies compared the brain activation between different stimulation conditions (e.g., unipedal versus bipedal stimulation, active versus passive movement) [22, 24–30, 32, 35–38, 40, 41, 43, 44], one between the condition of using mechanical stimulation induced by the device only and the motor imagery task only [23], one between stroke cohort and age-matched healthy counterparts [33] under the same stimulation condition, one between movements with and without the device stimulation [42], and the other six perform the comparison between task and blank blocks [20, 21, 31, 34, 39, 45].

### The fMRI observations

The 26 included fMRI studies characterized the supraspinal activation in response to somatosensory stimulation, to locomotor-like movements of the lower extremities, or to locomotor-like movements of the lower extremities in combination with extra simultaneous somatosensory stimulation. Key fMRI results are presented in Table 4.

The activation of supraspinal regions in response to somatosensory stimulation, as compared to rest, were assessed in five studies [20, 22–24, 46]. Activation was observed in cortical regions, including primary sensorimotor cortex (S1/M1) (number of studies (n) = 4) [20–23], the secondary sensory cortex (S2) (*n* = 2) [20, 23], the supplementary motor area (SMA) (*n* = 4) [21–24], the pre-motor cortex (PMC) (*n* = 1) [22], the prefrontal cortex (PFC) (*n* = 2) [22, 24], and insula (*n* = 2) [21, 22], as well as in subcortical regions including the basal ganglia (*n* = 2) [22, 24], the thalamus (*n* = 1) [24] and the cerebellum (Cb) (*n* = 1) [22]. Specifically, Hao et al., observed that the single-point sinusoidal stimulation applied to foot soles induced the activation in S1,S2 and M1 [20]. By using a newer version of the stimulation system (i.e., dual-drive foot-sole stimulator), the same group confirmed such results and further demonstrated that the insula and cingulate cortex may play important role in the processing of gait-related somatosensory stimuli [21]. In the three studies using the Korvit boot system, Kremneva et al., observed the involvement of PFC in response to standing-related stimulation and a broad involvement of the primary and secondary sensorimotor cortices in response to slow-walking stimulation [22]. In another study, Labriffe et al., observed that both gait-like and non-gait-like stimulation can induce similar-amplitude activation of the primary sensorimotor cortex and secondary somatosensory cortex bilaterally [23]. Using the same device, Jeanvoine et al., further observed that older age was associated with greater activation of right pre-SMA and mid-dorsolateral PFC [24].

The activation of supraspinal regions in response to locomotor-like movements of the lower extremities were assessed in 21 studies. In seventeen of these studies, single-joint movement of ankle (*n* = 6) [25–30] or multiple-joint movements (*n* = 11) [31–36, 38, 39, 42, 44, 45] were implemented, and in the other four, the locomotor-like movements were applied simultaneously with somatosensory stimuli as controlled by the device (i.e., combined-type stimulation) [37, 40, 41, 43]. Across these studies, activation was observed within the S1/M1 and S2 (*n* = 4) [29, 30, 39, 40], SMA (*n* = 15) [25, 27, 28, 30–34, 37, 39, 40, 42–45], PMC (*n* = 6) [29–31, 33, 34, 38], insula (*n* = 1) [26], basal ganglia (*n* = 6) [25, 29, 30, 34, 35, 39], thalamus (*n* = 2) [25, 30] and/or Cb (*n* = 15) [25, 27–37, 43–45] (Table 4).

With respect to single-joint movements, Trinastic et al. [25], and Newton et al. [30], compared the brain activation in response to active (i.e., the participant performed the movement voluntarily) ankle dorsiflexion and plantarflexion. Both studies observed that the S1/M1 and SMA were activated by both movements, and uniquely, other brain regions (e.g., right putamen) were activated only by dorsiflexion. Doolittle et al. [29], compared the activation in response to active unilateral and bilateral motions of the ankle joint, and observed that the activation was not significantly different between these two conditions. In another study [26], the participant was asked to complete one trial each of plantarflexion of left foot, right foot, or both feet against 15% of his/her maximum voluntary contraction lasting for 5 s. It was observed that compared to unilateral (i.e., left or right foot) plantarflexion, bilateral plantarflexion induced greater activation in multiple cortical and subcortical regions (e.g., S1/M1 and PMC). Two studies directly compared the brain activation in response to active and “passive” (i.e., the device imposed participant movement) ankle movement. The results of both studies suggested that both active and passive movement induced activation in sensorimotor and supplementary motor regions, and active movements uniquely induced activation in thalamic, frontal and cingulated regions, while passive movements induced activation in temporal and parietal areas [27, 28].

With respect to multiple-joint movements, Promjunyakul et al. [33], completed a study consisting of people recovering from stroke. They observed that as compared to age-matched healthy controls, the activation induced by actively pedaling, especially in the SMA, PMA and Cb, was significantly lower in people recovering from stroke. Brain activation in response to active and passive multiple-joint movements was also examined in four studies, showing different observations. Jaeger et al. [39], observed that compared to passive movements, active movements induced higher activation of S1/M1, SMA-proper, cingulate motor area, S2, Cb and putamen; in another study, Mehta et al., observed that compared to passive movements, active movements induced higher activation within Cb only [32]; Hollnagel et al., observed [38] that active movements elicited activation in a more extensive sensorimotor network; and Takahiro et al. [44], observed that the sensory regions of the brain as activated by passive movement was broader than the areas activated by active movement. Additionally, the association between the walking-related supraspinal activation and the parameters of the movement (i.e., pace or frequency of the motion) was examined in three studies. Specifically, Mehta et al. [32], reported that higher pedaling rate was associated with greater activation in S1/M1, SMA, and Cb; Toyomura et al. [36], showed that slower speed of stepping elicited more-extensive activity in sensorimotor cortex and Cb; and Martinez et al. [35], observed that the increase of stepping frequency was associated with a decrease in the activation of Cb.

With respect to the combined-type stimulation (i.e., locomotor-like movements applied simultaneously with somatosensory stimuli as controlled by the device), Ikeda et al. [43], observed higher level of activation within the motor regions and Cb as induced by the locomotor-like movements alone, as compared to the combined-type stimuli. Likewise, Hollnagel et al. [37], reported observations in one participant that the combined-type stimuli induced lower activation in SMA and PMC as compared to locomotor-like movements alone. In another study, Jaeger et al. [41], examined supraspinal activation when participants were performing stepping at different levels of force simulating the GRF experienced during real walking (i.e., 0% (no force), 20% and 40% of the individual’s body weight). No significant difference of the activation in sensorimotor regions between different levels of force was observed. In the same study, the influences of active and passive movements were also compared. It was observed that across all levels of force applied to the foot soles, active movements induced higher activation in Cb compared to passive movements. In another study from the same team, it was observed that both active and passive stepping movements with a load of 40% of individual’s body weight induce activation in the bilateral S1/M1, SMA-proper and Cb [40].

## Discussion

Thirteen different devices have been developed and implemented in fMRI studies. These previous works suggest a great promise to utilize these devices within the MRI environment to apply controlled walking-related stimuli to induce activation within multiple cortical and subcortical regions. Replication of results in many of the studies, however, was limited by small sample sizes, heterogeneity of the participant characteristics, and limited reporting of device validation, design, and fMRI compatibility. Future studies with larger sample size and rigorous design and validation testing are thus warranted to confirm and extend the observations from these publications on the characteristics of supraspinal control of gait in aging process and under the influences of age-related conditions via the implementation of these MRI-compatible devices.

In the included publications, device validation efforts have been made for stimulation performance of the devices, which are critical to the quality of neuroimaging data and the observations of brain activation. Several teams implemented adaptive control protocols based upon actuator performance [28], and/or utilized a iterative learning controller [38], to overcome the potential delay of work commands caused by the distance between the control unit (placed outside the scan room) and execution circuit [28, 48]. Future work (on pneumatic devices specifically) should consider working to reduce air loss in transmission via better sealing of the pistons, which would help to increase power and energy efficiency [49]. Additionally, between-subject variance in the characteristics of the lower extremity (e.g., different strength and lengths of the lower extremity between participants) may influence the device performance across individuals; and in turn, the degree to which a given device accurately simulates one or more elements of walking is a critical factor influencing fMRI results [21, 24]. It is thus recommended that future efforts to develop devices that can provide person-specific stimulation to more closely mimic their unique sensory or sensorimotor experience of walking, with the help of independently-controlled pneumatic actuators combined with pressure sensors to provide real-time feedback of applied pressure (i.e., closed-loop control of the stimulation) [21].

Efforts have also been made for the fMRI compatibility of these devices. For example, the execution units, which were placed inside the scan room, were uniquely manufactured with nonmagnetic and nonconductive materials with the goal to ensure sufficient robustness and to produce accurate and reliable stimulation to the individual during the MRI. Another challenge of using functional MRI to study brain activation induced by lower extremity stimulation is that stimulating or moving the legs and/or feet is likely to induce head motion artifact, which may in turn diminish image quality. Included studies successfully restricted movements of the head and trunk by using foam blocks and straps, and/or placing the knees in a flexed position and supporting the leg with a platform or cushion [26, 30, 34, 35]. Advanced signal processing techniques were also implemented to mitigate the influence of motion artifact on data quality in some studies. One commonly-used technique was “sparse sampling imaging” [50], which has been proven to effectively minimize movement-related artifacts on image quality [31–33, 39]. With the help of these strategies, studies that have reported the result of head-motion artifacts showed that the artifacts were less than 3 mm (in some cases even < 1 mm), which would not alter the observation of the brain activation [51]. It should be noted that most included studies only recruited healthy younger adult volunteer without overt neurological disorders (only one included individuals recovering from stroke), and the device performance and fMRI compatibility were not explicitly reported in seven of the included devices. It is thus worthwhile to explore and establish a thorough and standardized procedure to examine the device performance and MRI compatibility, and a validated protocol of data acquisition and processing, enabling the production of reproducible sensorimotor stimulation, and thus reliable observation of the brain’s activation across individuals [38, 48].

In general, across all of the included studies, fMRI results primarily linked walking-related sensorimotor stimulation to activation of S1/M1, SMA and Cb. These observations are not entirely aligned with those of previous neuroimaging studies using mental imagery of gait [23, 52–54]. Uniquely, Labriffe et al., directly compared the activation of supraspinal regions in response to mental imagery of gait and gait-related foot stimulation in a group of healthy younger adults. It was observed that mechanical stimulation, as compared to imagery, induced significantly greater activation in multiple regions (e.g., bilateral S1/M1 (especially areas related to lower limb), insula and Cb). These observations thus highlight the value of implementing MRI-compatible devices to apply walking-related stimuli to the lower extremities that mimics real walking, which can provide novel insights into the supraspinal sensorimotor control of gait that cannot be otherwise fully captured. Moreover, the stimulation and/or movements created by the different types of devices included in this review (e.g., foot sole somatosensory stimuli, stepping, pedaling, etc.), often induced different patterns in terms of negative and positive task associations, as well as recruitment of different brain regions. Therefore, in future studies, the design and characteristics of stimuli type appears to be critical and should be carefully taken into consideration.

The level of neuroimaging evidence of the activation of the brain in response to the stimulation was relatively low due to small sample sizes of participants and high heterogeneity of participant characteristics within and across studies. For example, several pilot studies focused primarily upon healthy younger adults or those with poorly-defined or under-reported inclusion and exclusion criteria. On the other hand, available evidence suggests that it is in fact feasible to simulate different sensory and/or motor aspects of walking within the MRI scanner without interfering substantially with imaging quality. Additionally, some important details were missing in several of the included publications, such as the utilization of data processing techniques, which is critical to the fMRI results. For example, eight of the included studies did not report if they completed the removal of head motion artifact [28, 37, 38, 41–45].

## Conclusion

In conclusion, this review comprehensively summarized the current research and development achievements of the MRI-compatible devices that simulate and provide the walking-related sensorimotor stimulation when people lie motionless during the MRI scan, and thus enable the characterization of the supraspinal sensorimotor control of walking (e.g., brain’s responsiveness) via fMRI. The evidence provided by these publications suggest that it is promising to implement these devices in human study to help characterize the supraspinal control of walking. However, the small sample size of participants and varied study design limited the power of evidence. Therefore, well-powered studies with more rigorous study protocols are warranted to confirm the feasibility of implementing these devices in different populations and those preliminary observations reported here, facilitate comparison between studies, and ultimately, elucidate the supraspinal networks involved in control pertaining to the regulation of gait in relatively-healthy cohorts and in those suffering from age- or disease-related gait disorders [55, 56].

## Data Availability

The dataset supporting the conclusions of this article is included within the article.

## Abbreviations

MRI: Magnetic Resonance Imaging
fMRI: functional Magnetic Resonance Imaging
Bra.Di.P.O.: Brain Discovery Pneumatic Orthosis
pseudogait-MRCD: Pseudogait-Magnetic Resonance Compatible Device
MARCOS: Magnetic Resonance Compatible Stepper
LOMS: Lower-extremity Motion Simulator
PVC: Polyvinyl Chloride
SNR: Signal-to-Noise Ratio
SFNR: Signal-to-Fluctuation-Noise Ratio
S1/M1: Primary Sensorimotor Cortex
S2: Secondary Sensory Cortex
SMA: Supplementary Motor Area
PMC: Pre-motor Cortex
PFC: Prefrontal Cortex
Cb: Cerebellum
